# Supplementary material for: Class-Specific Effects of ARBs Versus ACE Inhibitors on Survival and Cardiovascular Outcomes in MASLD
Source: Int J Mol Sci. 2025 Oct 16;26(20):10061. doi: 10.3390/ijms262010061 (PMC12563377; doi:10.3390/ijms262010061)
Supplement: Supplementary file 1 [file ijms-26-10061-s001.zip › ijms-3866504-supplementary.pdf]

Supplementary Information

Class-specific effects of ARBs versus ACE Inhibitors on sur-vival and cardiovascular outcomes in MASLD

Tom Ryu [1]†, Yeon Joo Seo[2]†, Jaejun Lee[2], Ji Won Han[2], Hyun Yang[2], and Keungmo Yang[2]*
†Tom Ryu and Yeon Joo Seo contributed to this work equally.

[1]Department of Internal Medicine, Institute for Digestive Research, Digestive Disease Center, Soonchunhyang University College of Medicine, Seoul 04401, Republic of Korea
[2]Department of Internal Medicine, Division of Gastroenterology and Hepatology, College of Medicine, The Catholic University of Korea, Seoul 06591, Republic of Korea

ADDRESS CORRESPONDENCE AND REPRINT REQUESTS TO:
Keungmo Yang, M.D., Ph.D., Division of Gastroenterology and Hepatology, Department of Internal Medicine, College of Medicine, The Catholic University of Korea, 222, Banpo-daero, Seocho-gu, Seoul, 06591, Republic of Korea, Tel: +82-2-2258-2073 FAX: +82-2-3481-4025, E-mail: yang27jin@catholic.ac.kr

Keywords: metabolic dysfunction-associated steatotic liver disease, angiotensin II receptor blockers, angiotensin converting enzyme inhibitors, survival, cardiovascular event

Supplementary Figures

- **Supplementary Fig. S1.** Decrease of SMDs after IPTW in the entire cohort, No SLD cohort and MASLD cohort.
- **Supplementary Fig. S2.** Kaplan-Meier curves of ARB and ACEI users for all-cause mortality in the entire cohort before and after IPTW.
- **Supplementary Fig. S3.** Kaplan-Meier curves of ARB and ACEI users for incidence of cardiovascular event, decompensation event, and HCC in the entire cohort.
- **Supplementary Fig. S4.** Kaplan-Meier curves of ARB and ACEI users for incidence of cardiovascular event in the MASLD and No SLD cohort.
- **Supplementary Fig. S5.** Kaplan-Meier curves of ARB and ACEI users for incidence of decompensation event and HCC in the MASLD and No SLD cohort.
- **Supplementary Fig. S6.** Kaplan-Meier curves for all-cause mortality and incidence of cardiovascular events of ARB and ACEI users, with or without significant liver fibrosis in the entire cohort after IPTW.
- **Supplementary Fig. S7.** Kaplan-Meier curves for all-cause mortality and incidence of cardiovascular events of ARB and ACEI users, with or without significant liver fibrosis in the No SLD cohort after IPTW.
- **Supplementary Fig. S8.** Kaplan-Meier curves for all-cause mortality and incidence of cardiovascular events of ARB and ACEI users, with rule-in thresholds of NFS and FIB-4 in the MASLD cohort after IPTW.
- **Supplementary Fig. S9.** Spatial transcriptomic expression of ACEI- and ARB-related genes in mouse liver.

Supplementary Figure 1

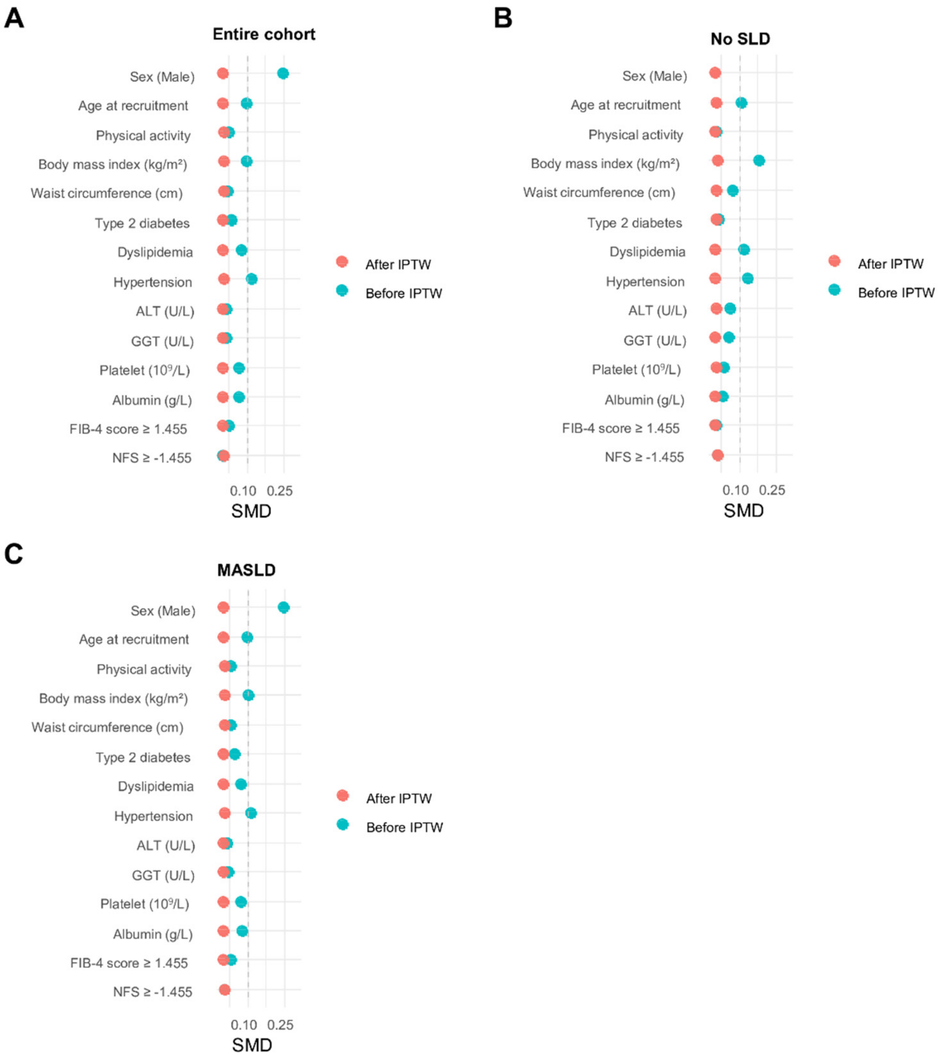

Supplementary Fig. S1. Decrease of SMDs after IPTW in the entire cohort, No SLD cohort and MASLD cohort. (A) Entire cohort. (B) No SLD cohort. (C) MASLD. IPTW, inverse probability of treatment weighting; SMD, standardized mean difference; SLD, steatotic liver disease; MASLD, metabolic dysfunction-associated steatotic liver disease.

Supplementary Figure 2

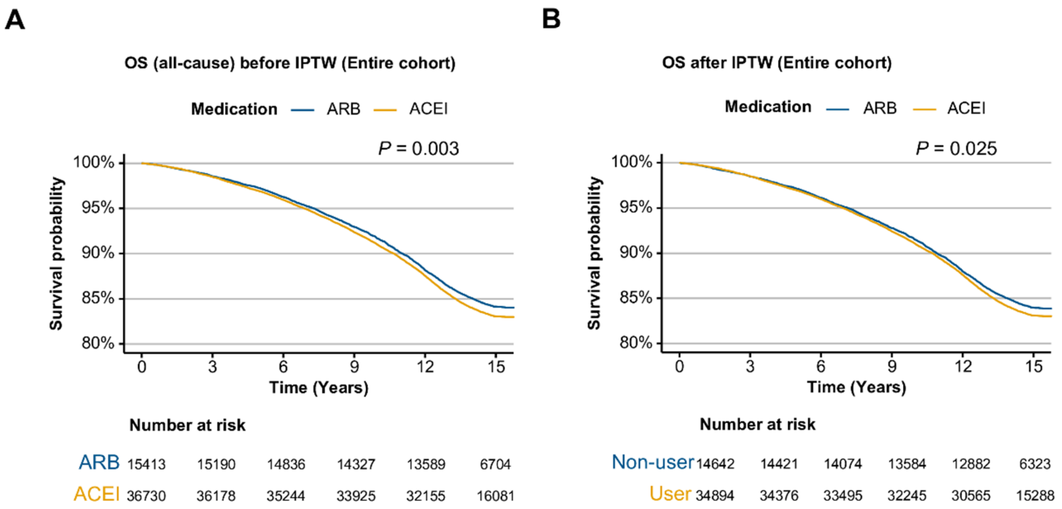

Supplementary Fig. S2. Kaplan-Meier curves of ARB and ACEI users for all-cause mortality in the entire cohort before and after IPTW. (A) Before IPTW. (B) After IPTW. ARB, angiotensin II receptor blocker; ACEI, angiotensin-converting enzyme inhibitor; OS, overall survival; IPTW, inverse probability of treatment weighting.

Supplementary Figure 3

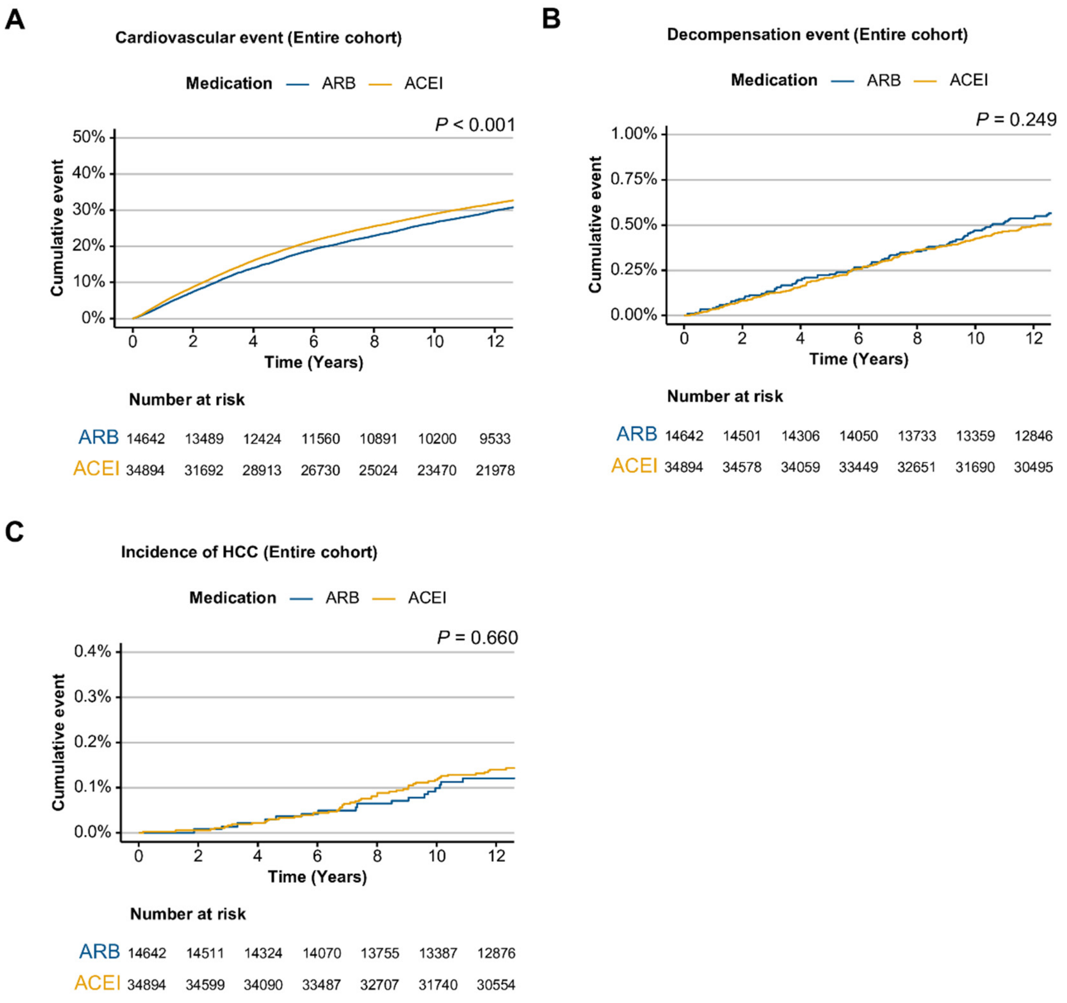

Supplementary Fig. S3. Kaplan-Meier curves of ARB and ACEI users for incidence of cardiovascular event, decompensation event, and HCC in the entire cohort after IPTW. (A) Incidence of cardiovascular event. (B) Incidence of decompensation event. (C) Incidence of HCC. ARB, angiotensin II receptor blocker; ACEI, angiotensin-converting enzyme inhibitor; HCC, hepatocellular carcinoma; IPTW, inverse probability of treatment weighting.

Supplementary Figure 4

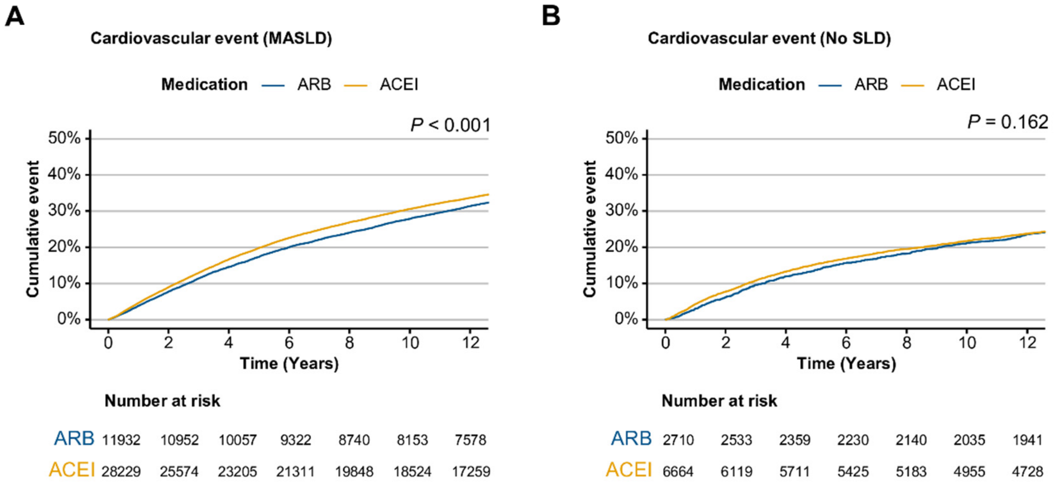

Supplementary Fig. S4. Kaplan-Meier curves of ARB and ACEI users for incidence of cardiovascular event in the MASLD and No SLD cohort after IPTW. (A) MASLD. (B) No SLD. ARB, angiotensin II receptor blocker; ACEI, angiotensin-converting enzyme inhibitor; MASLD, metabolic dysfunction-associated steatotic liver disease; SLD, steatotic liver disease; IPTW, inverse probability of treatment weighting.

Supplementary Figure 5

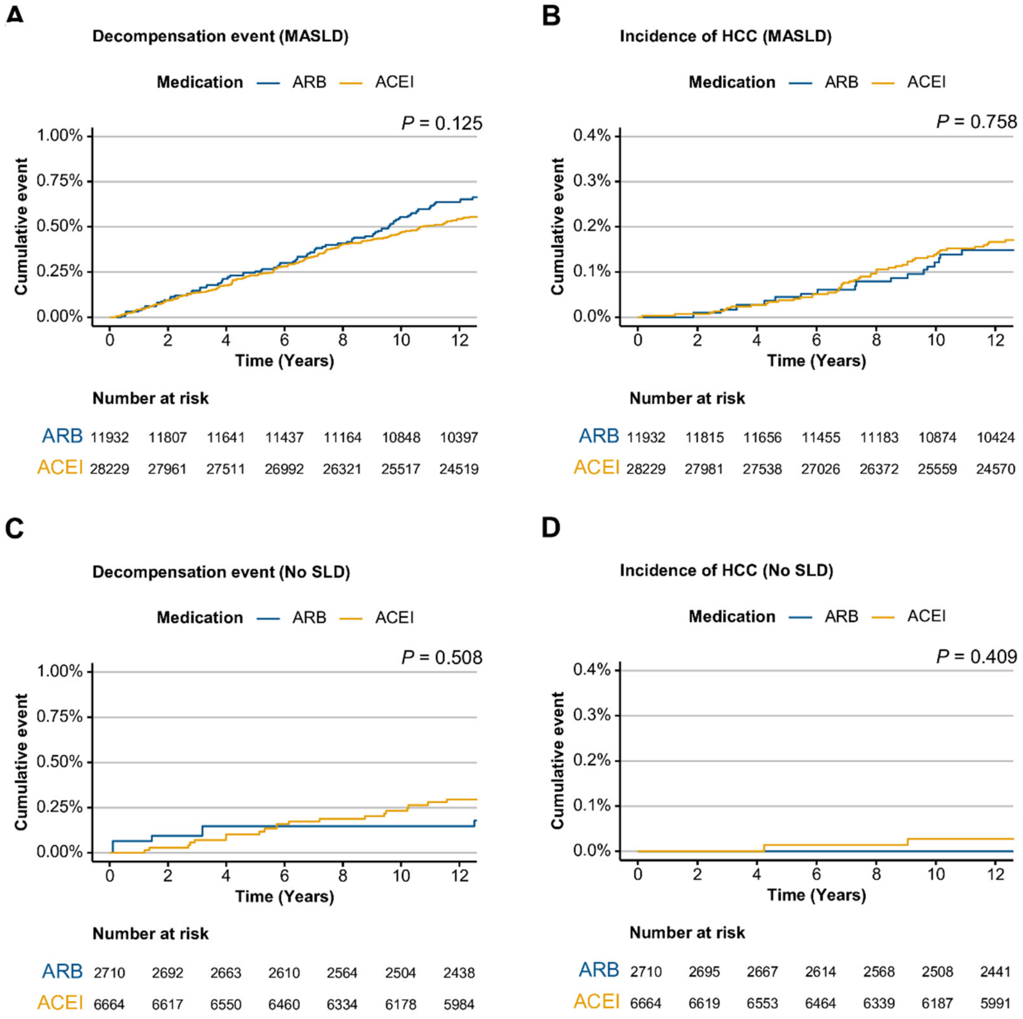

Supplementary Fig. S5. Kaplan-Meier curves of ARB and ACEI users for incidence of decompensation event and HCC in the MASLD and No SLD cohort after IPTW. (A) Incidence of decompensation event in the MASLD. (B) Incidence of HCC in the MASLD. (C) Incidence of decompensation event in the No SLD. (D) Incidence of HCC in the No SLD. ARB, angiotensin II receptor blocker; ACEI, angiotensin-converting enzyme inhibitor; MASLD, metabolic dysfunction-associated steatotic liver disease; SLD, steatotic liver disease; HCC, hepatocellular carcinoma; IPTW, inverse probability of treatment weighting.

Supplementary Figure 6

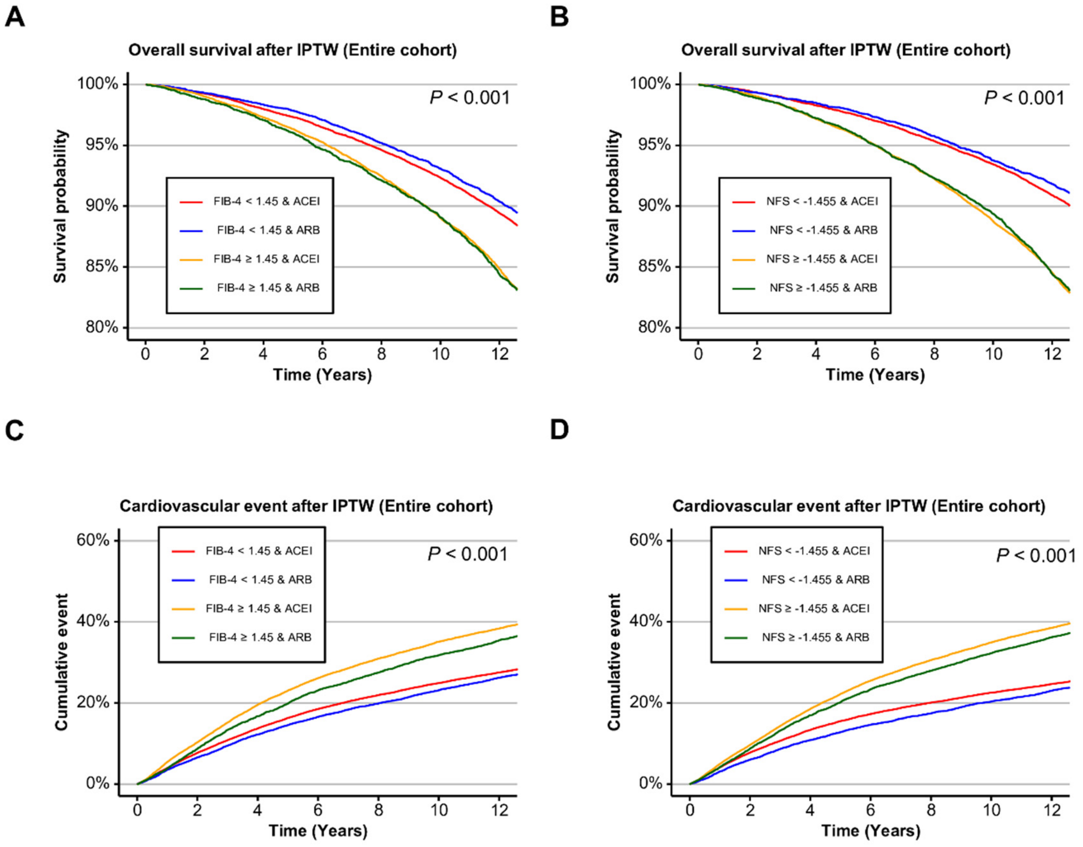

Supplementary Fig. S6. Kaplan-Meier curves for all-cause mortality and incidence of cardiovascular events of ARB and ACEI users, with or without significant liver fibrosis in the entire cohort after IPTW. (A) all-cause mortality with FIB-4 < 1.45 or ≥ 1.45. (B) all-cause mortality with NFS < -1.455 or ≥ -1.455. (C) Cardiovascular event incidence users with FIB-4 < 1.45 or ≥ 1.45. (D) Cardiovascular event incidence with NFS < -1.455 or ≥ -1.455. ARB, angiotensin II receptor blocker; ACEI, angiotensin-converting enzyme inhibitor; IPTW, inverse probability of treatment weighting; FIB-4, fibrosis-4; NFS, non-alcoholic fatty liver disease fibrosis score.

Supplementary Figure 7

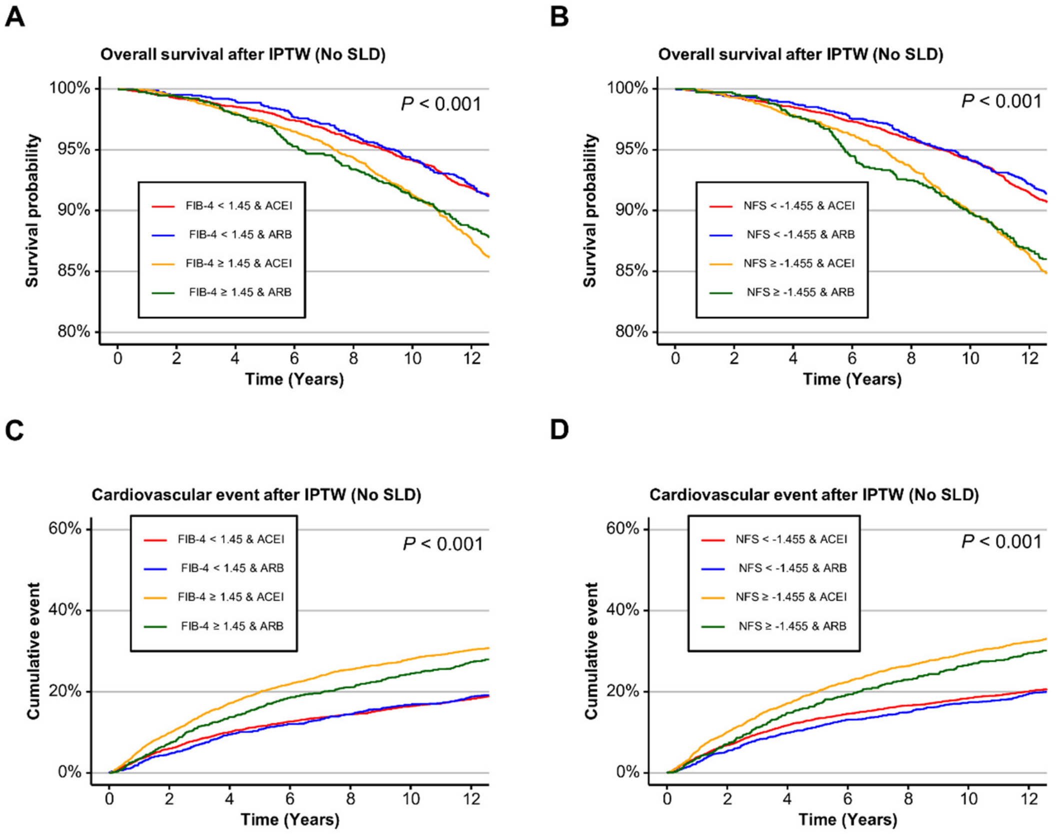

Supplementary Fig. S7. Kaplan-Meier curves for all-cause mortality and incidence of cardiovascular events of ARB and ACEI users, with or without significant liver fibrosis in the No SLD cohort after IPTW. (A) all-cause mortality with FIB-4 < 1.45 or ≥ 1.45. (B) all-cause mortality with NFS < -1.455 or ≥ -1.455. (C) Cardiovascular event incidence users with FIB-4 < 1.45 or ≥ 1.45. (D) Cardiovascular event incidence with NFS < -1.455 or ≥ -1.455. ARB, angiotensin II receptor blocker; ACEI, angiotensin-converting enzyme inhibitor; IPTW, inverse probability of treatment weighting; FIB-4, fibrosis-4; NFS, non-alcoholic fatty liver disease fibrosis score; SLD, steatotic liver disease.

Supplementary Figure 8

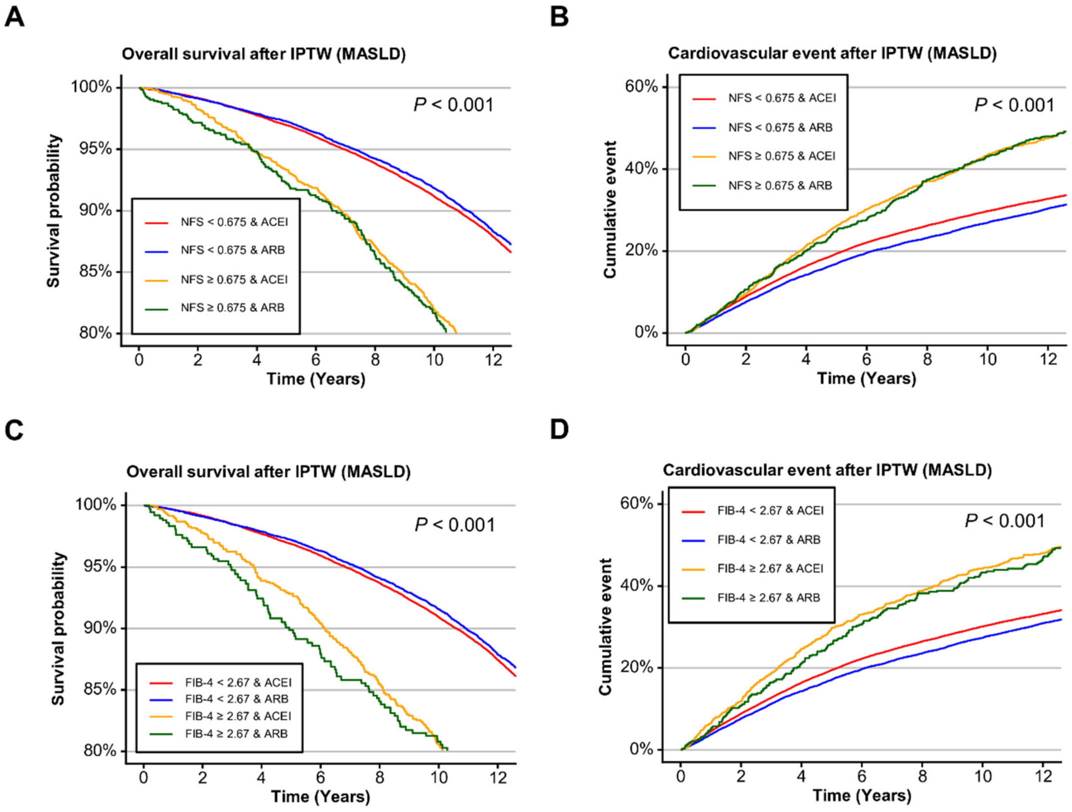

Supplementary Fig. S8. Kaplan-Meier curves for all-cause mortality and incidence of cardiovascular events of ARB and ACEI users, with rule-in thresholds of NFS and FIB-4 in the MASLD cohort after IPTW. (A) all-cause mortality with NFS < 0.675 or ≥ 0.675. (B) cardiovascular event incidence with NFS < 0.675 or ≥ 0.675. (C) all-cause mortality with FIB-4 < 2.67 or ≥ 2.67. (D) Cardiovascular event incidence with FIB-4 < 2.67 or ≥ 2.67. ARB, angiotensin II receptor blocker; ACEI, angiotensin-converting enzyme inhibitor; IPTW, inverse probability of treatment weighting; FIB-4, fibrosis-4; NFS, non-alcoholic fatty liver disease fibrosis score; MASLD, metabolic dysfunction-associated steatotic liver disease.

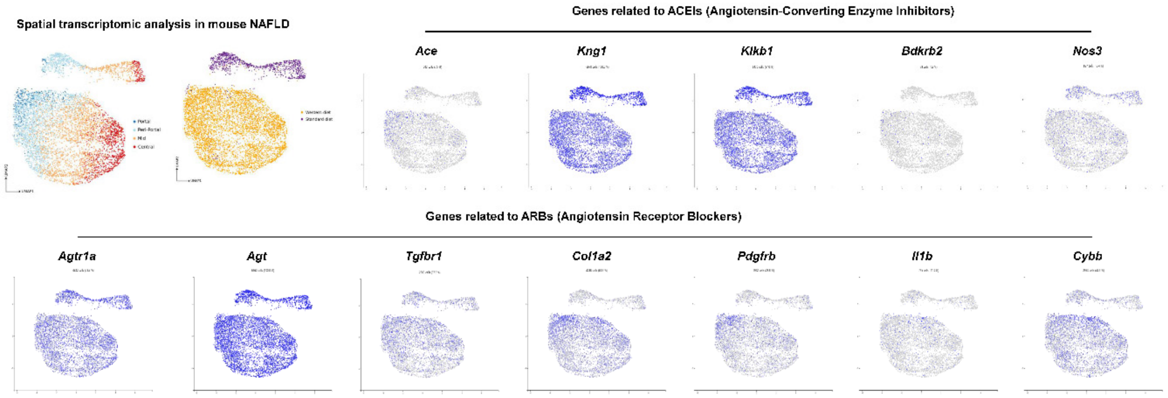

Supplementary Fig. S9. Spatial transcriptomic expression of ACEI- and ARB-related genes in mouse liver. Expression of ACEI-related genes (*Ace, Kng1, Klkb1, Bdkrb2, Nos3*) was generally low with minimal dietary changes, while ARB-related genes (*Agtr1a, Agt, Tgfbr1, Col1a2, Pdgfrb, Il1b, Cybb*) were more highly expressed and upregulated in Western diet (yellow) compared to standard diet (purple).

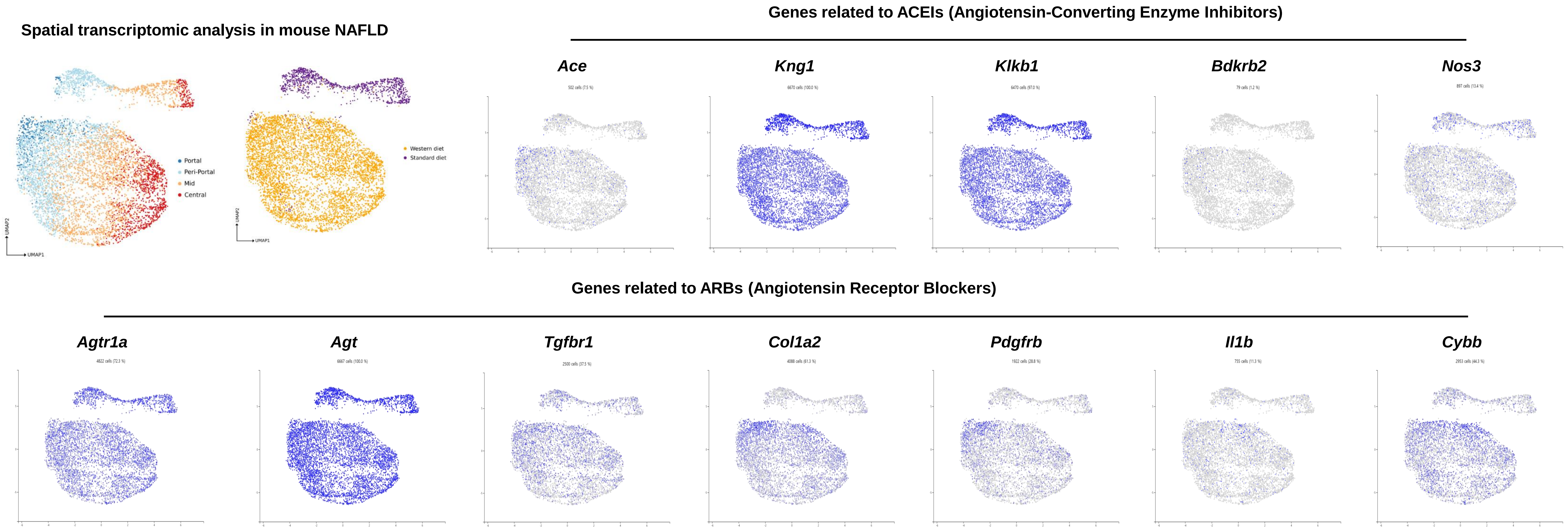

Supplementary Fig. S9. Spatial transcriptomic expression of ACEI- and ARB-related genes in mouse liver. Expression of ACEI-related genes (*Ace*, *Kng1*, *Klkb1*, *Bdkrb2*, *Nos3*) was generally low with minimal dietary changes, while ARB-related genes (*Agtr1a*, *Agt*, *Tgfbr1*, *Col1a2*, *Pdgfrb*, *Il1b*, *Cybb*) were more highly expressed and upregulated in Western diet (yellow) compared to standard diet (purple).

Supplementary Tables

- **Supplementary Table S1.** Medication codes sourced from UK Biobank (Data-Field 20003).
- **Supplementary Table S2.** Relative ratios based on medications.
- **Supplementary Table S3.** Baseline clinical characteristics of the entire cohort before and after IPTW.
- **Supplementary Table S4.** Baseline clinical characteristics before IPTW.
- **Supplementary Table S5.** Univariate and multivariate analysis for overall survival before and after IPTW in the entire cohort.
- **Supplementary Table S6.** Univariate and multivariate analyses of overall survival before and after IPTW in participants without SLD.
- **Supplementary Table S7.** Overall survival comparison between ARB and ACEI users after IPTW adjustment.
- **Supplementary Table S8.** Comparison of overall survival between ARB and ACEI after IPTW: subgroup analyses by sex, age, body mass index, and type 2 diabetes.
- **Supplementary Table S9.** Comparison of cardiovascular events between ARB and ACEI after IPTW: subgroup analyses by sex, age, body mass index, and type 2 diabetes.
- **Supplementary Table S10.** IPTW-adjusted comparison of overall survival between ARB and ACEI across subgroups defined by hepatic fibrosis score.
- **Supplementary Table S11.** Supplementary Table 10. IPTW-adjusted comparison of cardiovascular events between ARB and ACEI across subgroups defined by hepatic fibrosis score.

Supplementary Table S1. Medication codes sourced from UK Biobank (Data-Field 20003).

ARB	ACEI
1140916356	1140860696
1141179974	1140864952
1141151018	1140864910
1141151016	1140860714
1141145660	1140860728
1141201038	1140860736
1141145668	1140881706
1141152998	1140860750
1141153006	1140860758
1141172686	1140860764
1141172682	1140888552
1141156836	1140860790
1141156846	1140860806
1141166006	1141188408
1141187788	1140860882
1141172492	1140860904
1141187790	1140888556
1141171336	1140888560
1141171344	1141180592
1141193282	1141180598
1141193346	1141164148
	1141164154

ARB, angiotensin receptor blocker; ACEI, angiotensin-converting enzyme inhibitor.

Supplementary Table S2. Relative ratios based on medications.

Group	Medications	Number of participants	Relative ratio (%)
**Overall**	**ARB**	15,413	29.6
	**ACEI**	36,730	70.4
**No SLD**	**ARB**	2,853	28.9
	**ACEI**	7,015	71.1
**MASLD**	**ARB**	12,560	29.7
	**ACEI**	29,715	70.3
**Male**	**ARB**	8,410	35.1
	**ACEI**	15,554	64.9
**Female**	**ARB**	7,003	24.9
	**ACEI**	21,176	75.1
**Age < 65**	**ARB**	10,140	28.9
	**ACEI**	24,999	71.1
**Age ≥ 65**	**ARB**	5,273	31.0
	**ACEI**	11,731	69.0

ARB, angiotensin receptor blocker; ACEI, angiotensin-converting enzyme inhibitor; SLD, steatotic liver disease; MASLD, metabolic dysfunction-associated steatotic liver disease.

Supplementary Table S3. Baseline clinical characteristics of the entire cohort before and after IPTW.

Clinical characteristics	Before IPTW			After IPTW		
	Medication		SMD	Medication		SMD
	ARB, N = 15,413	ACEI, N = 36,730		Non-user, N = 14,642	User, N = 34,894	
**Sex (Male)**	7,003 (45.4%)	21,176 (57.7%)	0.246	26,744 (54.0%)	7,894 (53.9%)	0.002
**Age at recruitment**	60.90 ± 6.35	60.25 ± 6.69	0.099	60.44 ± 6.60	60.44 ± 6.60	0.001
**Physical activity**			0.028			0.003
More than 4 times	11,852 (76.9%)	28,672 (78.1%)		38,459 (77.6%)	11,353 (77.5%)	
Under 4 times	3,561 (23.1%)	8,058 (21.9%)		11,077 (22.4%)	3,289 (22.5%)	
**Body mass index (kg/m^2)**	30.33 ± 5.60	29.79 ± 5.54	0.097	29.97 ± 5.58	30.00 ± 5.36	0.006
**Waist circumference (cm)**	97.46 ± 14.31	97.73 ± 14.32	0.018	97.69 ± 14.34	97.73 ± 14.07	0.004
**Type 2 diabetes**	4,552 (29.5%)	11,456 (31.2%)	0.036	15,230 (30.7%)	4,512 (30.8%)	0.002
**Dyslipidemia**	9,872 (64.0%)	24,863 (67.7%)	0.077	33,002 (66.6%)	9,757 (66.6%)	< 0.001
**Hypertension**	15,220 (98.7%)	35,658 (97.1%)	0.117	48,344 (97.6%)	14,296 (97.6%)	0.004
**ALT (U/L)**	25.70 ± 14.46	25.93 ± 15.14	0.015	25.89 ± 15.31	25.89 ± 14.20	< 0.001
**GGT (U/L)**	42.07 ± 43.22	42.79 ± 42.81	0.017	42.61 ± 43.02	42.63 ± 43.49	0.001
**Platelet (10^9/L)**	254.78 ± 62.74	250.64 ± 62.71	0.066	251.89 ± 62.75	251.93 ± 62.54	0.001
**Albumin (g/L)**	4.50 ± 0.26	4.52 ± 0.26	0.069	4.51 ± 0.26	4.51 ± 0.26	0.002
**FIB-4 score ≥ 1.45**	6,014 (39.0%)	14,814 (40.3%)	0.027	19,797 (40.0%)	5,856 (40.0%)	0.001
**NFS ≥ -1.455**	8,008 (52.0%)	19,083 (52.0%)	< 0.001	25,770 (52.0%)	7,631 (52.1%)	0.003
**ASCVD risk score**	14.04 ± 12.50	14.49 ± 12.92	0.036	14.40 ± 12.92	14.31 ± 12.77	0.006

Data are described as mean ± standard deviation or n (%). IPTW, inverse probability of treatment weighting; ARB, angiotensin receptor blocker; ACEI, angiotensin-converting enzyme inhibitor; ALT, alanine aminotransferase (upper normal limit = 40 U/L); GGT, gamma-glutamyl transferase (upper normal limit = 50 U/L); SMD, standardized mean difference; FIB-4, fibrosis-4; NFS, NAFLD fibrosis score; ASCVD, atherosclerotic cardiovascular disease.

Supplementary Table S4. Baseline clinical characteristics before IPTW.

Clinical characteristics	No SLD			MASLD		
	Medication		SMD	Medication		SMD
	ARB, N = 2,853	ACEI, N = 7,015		ARB, N = 12,560	ACEI, N = 29,715	
**Sex (Male)**	634 (22.2%)	2,509 (35.8%)	0.302	6,369 (50.7%)	18,667 (62.8%)	0.246
**Age at recruitment**	61.23 ± 6.34	60.52 ± 6.67	0.108	60.83 ± 6.35	60.19 ± 6.69	0.098
**Physical activity**			0.004			0.033
More than 4 times	2,411 (84.5%)	5,917 (84.3%)		9,441 (75.2%)	22,755 (76.6%)	
Under 4 times	442 (15.5%)	1,098 (15.7%)		3,119 (24.8%)	6,960 (23.4%)	
**Body mass index (kg/m^2)**	24.23 ± 2.30	23.82 ± 2.24	0.177	31.72 ± 5.18	31.20 ± 5.13	0.100
**Waist circumference (cm)**	79.46 ± 7.01	79.97 ± 7.19	0.072	101.55 ± 12.24	101.92 ± 12.22	0.030
**Type 2 diabetes**	330 (11.6%)	844 (12.0%)	0.014	2,064 (16.4%)	5,188 (17.5%)	0.044
**Dyslipidemia**	1,433 (50.2%)	3,938 (56.1%)	0.119	4,222 (33.6%)	10,612 (35.7%)	0.070
**Hypertension**	2,805 (98.3%)	6,745 (96.2%)	0.132	8,439 (67.2%)	20,925 (70.4%)	0.113
**ALT (U/L)**	18.54 ± 7.35	19.02 ± 7.89	0.062	12,415 (98.8%)	28,913 (97.3%)	0.015
**GGT (U/L)**	23.59 ± 14.79	24.41 ± 15.08	0.055	27.33 ± 15.17	27.56 ± 15.96	0.019
**Platelet (10^9/L)**	254.65 ± 61.01	252.42 ± 64.09	0.036	46.26 ± 46.34	47.13 ± 45.97	0.073
**Albumin (g/L)**	4.52 ± 0.27	4.53 ± 0.26	0.029	254.81 ± 63.12	250.22 ± 62.37	0.078
**FIB-4 score ≥ 1.45**	1,373 (48.1%)	3,401 (48.5%)	0.007	4.49 ± 0.26	4.51 ± 0.26	0.030
**NFS ≥ -1.455**	952 (33.4%)	2,314 (33.0%)	0.008	4,641 (37.0%)	11,413 (38.4%)	0.005
**ASCVD risk score**	6.88 ± 6.00	6.95 ± 6.24	0.011	15.67 ± 13.02	16.27 ± 13.44	0.036

Data are described as mean ± standard deviation or n (%). IPTW, inverse probability of treatment weighting; SLD, steatotic liver disease; MASLD, metabolic dysfunction-associated steatotic liver disease; ARB, angiotensin receptor blocker; ACEI, angiotensin-converting enzyme inhibitor; ALT, alanine aminotransferase (upper normal limit = 40 U/L); GGT, gamma-glutamyl transferase (upper normal limit = 50 U/L); SMD, standardized mean difference; FIB-4, fibrosis-4; NFS, NAFLD fibrosis score; ASCVD, atherosclerotic cardiovascular disease.

Supplementary Table S5. Univariate and multivariate analysis for overall survival before and after IPTW in the entire cohort.

Clinical characteristics	Univariate analysis			Multivariate analysis		
**Before IPTW**						
	**HR**	**95% CI**	***P* value**	**HR**	**95% CI**	***P* value**
**Sex (Male)**	1.62	1.55, 1.69	< 0.001	1.35	1.27, 1.43	< 0.001
**Age at recruitment**	1.09	1.09, 1.09	< 0.001	1.08	1.08, 1.09	< 0.001
**Physical activity (Under 4 times)**	1.22	1.16, 1.28	< 0.001	1.18	1.12, 1.24	< 0.001
**Body mass index (kg/m^2)**	1.02	1.01, 1.02	< 0.001	0.97	0.96, 0.98	< 0.001
**Waist circumference (cm)**	1.02	1.02, 1.02	< 0.001	1.02	1.02, 1.02	< 0.001
**Type 2 diabetes**	1.81	1.73, 1.89	< 0.001	1.53	1.45, 1.61	< 0.001
**Dyslipidemia**	1.57	1.49, 1.65	< 0.001	1.08	1.02, 1.14	0.004
**ALT (U/L)**	1.00	0.99, 1.00	< 0.001	0.99	0.99, 0.99	< 0.001
**GGT (U/L)**	1.00	1.00, 1.00	< 0.001	1.00	1.00, 1.00	< 0.001
**Platelet (10^9/L)**	1.00	1.00, 1.00	< 0.001	1.00	1.00, 1.00	< 0.001
**Albumin (g/L)**	0.40	0.37, 0.43	< 0.001	0.51	0.47, 0.55	< 0.001
**FIB-4 score ≥ 1.45**	1.51	1.45, 1.58	< 0.001	1.17	1.10, 1.24	< 0.001
**NFS ≥ -1.455**	1.87	1.79, 1.95	< 0.001	1.04	0.97, 1.11	0.263
**ASCVD risk score**	1.03	1.03, 1.03	< 0.001	1.01	1.00, 1.01	< 0.001
**ARB user**	0.93	0.89, 0.98	0.003	0.94	0.90, 0.98	0.009
**After IPTW**						
**Clinical characteristics**	**Univariate analysis**			**Multivariate analysis**		
	**HR**	**95% CI**	***P* value**	**HR**	**95% CI**	***P* value**
**Sex (Male)**	1.61	1.54, 1.69	< 0.001	1.36	1.28, 1.45	< 0.001
**Age at recruitment**	1.09	1.09, 1.10	< 0.001	1.08	1.08, 1.09	< 0.001
**Physical activity (Under 4 times)**	1.22	1.16, 1.28	< 0.001	1.18	1.12, 1.24	< 0.001
**Body mass index (kg/m^2)**	1.02	1.01, 1.02	< 0.001	0.97	0.96, 0.98	< 0.001
**Waist circumference (cm)**	1.02	1.02, 1.02	< 0.001	1.02	1.02, 1.02	< 0.001
**Type 2 diabetes**	1.81	1.73, 1.89	< 0.001	1.52	1.44, 1.61	< 0.001
**Dyslipidemia**	1.58	1.50, 1.66	< 0.001	1.08	1.03, 1.14	0.002
**ALT (U/L)**	1.00	0.99, 1.00	< 0.001	0.99	0.99, 0.99	< 0.001
**GGT (U/L)**	1.00	1.00, 1.00	< 0.001	1.00	1.00, 1.00	< 0.001
**Platelet (10^9/L)**	1.00	1.00, 1.00	< 0.001	1.00	1.00, 1.00	< 0.001
**Albumin (g/L)**	0.39	0.36, 0.43	< 0.001	0.50	0.46, 0.55	< 0.001
**FIB-4 score ≥ 1.45**	1.51	1.45, 1.58	< 0.001	1.17	1.11, 1.24	< 0.001
**NFS ≥ -1.455**	1.86	1.78, 1.95	< 0.001	1.04	0.97, 1.11	0.293
**ASCVD risk score**	1.03	1.03, 1.03	< 0.001	1.01	1.00, 1.01	< 0.001
**ARB user**	0.95	0.90, 0.99	0.021	0.94	0.90, 0.99	0.017

IPTW, inverse probability of treatment weighting; HR, hazard ratio; CI, confidence interval; ALT, alanine aminotransferase (upper normal limit = 40 U/L); GGT, gamma-glutamyl transferase (upper normal limit = 50 U/L); FIB-4, fibrosis-4; NFS, NAFLD fibrosis score; ASCVD, atherosclerotic cardiovascular disease; ARB, angiotensin receptor blocker.

Supplementary Table S6. Univariate and multivariate analyses of overall survival before and after IPTW in participants without SLD.

Clinical characteristics	Univariate analysis			Multivariate analysis		
**Before IPTW**						
	HR	95% CI	*P* value	HR	95% CI	*P* value
Sex (Male)	1.76	1.57, 1.96	< 0.001	1.28	1.10, 1.49	0.002
Age at recruitment	1.10	1.09, 1.11	< 0.001	1.08	1.07, 1.10	< 0.001
Physical activity (Under 4 times)	1.09	0.94, 1.26	0.242			
Body mass index (kg/m^2)	0.89	0.87, 0.91	< 0.001	0.87	0.84, 0.90	< 0.001
Waist circumference (cm)	1.02	1.01, 1.03	< 0.001	1.02	1.01, 1.04	< 0.001
Type 2 diabetes	1.94	1.69, 2.23	< 0.001	1.59	1.36, 1.87	< 0.001
Dyslipidemia	1.56	1.39, 1.75	< 0.001	1.17	1.03, 1.32	0.012
ALT (U/L)	1.00	0.99, 1.00	0.156			
GGT (U/L)	1.01	1.01, 1.01	< 0.001	1.01	1.01, 1.01	< 0.001
Platelet (10^9/L)	1.00	1.00, 1.00	0.699			
Albumin (g/L)	0.41	0.33, 0.50	< 0.001	0.53	0.43, 0.66	< 0.001
FIB-4 score ≥ 1.45	1.48	1.33, 1.66	< 0.001	1.05	0.90, 1.22	0.527
NFS ≥ -1.455	1.61	1.44, 1.79	< 0.001	1.13	0.96, 1.32	0.141
ASCVD risk score	1.07	1.06, 1.08	< 0.001	1.02	1.01, 1.03	0.002
ARB user	0.90	0.79, 1.01	0.081			
**After IPTW**						
**Clinical characteristics**	**Univariate analysis**			**Multivariate analysis**		
	HR	95% CI	*P* value	HR	95% CI	*P* value
Sex (Male)	1.79	1.60, 2.00	< 0.001	1.30	1.11, 1.51	0.001
Age at recruitment	1.10	1.08, 1.11	< 0.001	1.08	1.07, 1.10	< 0.001
Physical activity (Under 4 times)	1.06	0.91, 1.23	0.435			
Body mass index (kg/m^2)	0.89	0.87, 0.92	< 0.001	0.87	0.84, 0.90	< 0.001
Waist circumference (cm)	1.02	1.01, 1.03	< 0.001	1.02	1.01, 1.04	< 0.001
Type 2 diabetes	1.92	1.67, 2.20	< 0.001	1.58	1.33, 1.86	< 0.001
Dyslipidemia	1.58	1.40, 1.77	< 0.001	1.19	1.05, 1.35	0.006
ALT (U/L)	1.00	0.99, 1.00	0.245			
GGT (U/L)	1.01	1.01, 1.01	< 0.001	1.01	1.01, 1.01	< 0.001
Platelet (10^9/L)	1.00	1.00, 1.00	0.682			
Albumin (g/L)	0.39	0.32, 0.48	< 0.001	0.50	0.40, 0.63	< 0.001
FIB-4 score ≥ 1.45	1.48	1.32, 1.66	< 0.001	1.06	0.91, 1.23	0.449
NFS ≥ -1.455	1.58	1.42, 1.77	< 0.001	1.11	0.94, 1.31	0.218
ASCVD risk score	1.07	1.06, 1.07	< 0.001	1.02	1.00, 1.03	0.008
ARB user	0.98	0.86, 1.11	0.318			

IPTW, inverse probability of treatment weighting; HR, hazard ratio; CI, confidence interval; ALT, alanine aminotransferase (upper normal limit = 40 U/L); GGT, gamma-glutamyl transferase (upper normal limit = 50 U/L); FIB-4, fibrosis-4; NFS, NAFLD fibrosis score; ASCVD, atherosclerotic cardiovascular disease; ARB, angiotensin receptor blocker.

Supplementary Table S7. Overall survival comparison between ARB and ACEI users after IPTW adjustment.

Medication	Entire cohort				
	Number of patients	Number of events	Number of events / 100 patient-years	HR (95% CI)	*P* value
**ARB**	15,413	2,410	1.110	Reference	
**ACEI**	36,730	6,134	1.190	1.06 (1.01-1.11)	0.025
**Medication**	**No SLD**				
	**Number of patients**	**Number of events**	**Number of events / 100 patient-years**	**HR (95% CI)**	***P* value**
**ARB**	2,853	346	0.850	Reference	
**ACEI**	7,015	946	0.947	1.07 (0.94-1.21)	0.318
**Medication**	**MASLD**				
	**Number of patients**	**Number of events**	**Number of events / 100 patient-years**	**HR (95% CI)**	***P* value**
**ARB**	12,560	2,064	1.170	Reference	
**ACEI**	29,715	5,188	1.248	1.06 (1.01-1.12)	0.029

IPTW, inverse probability of treatment weighting; ARB, angiotensin receptor blocker; ACEI, angiotensin-converting enzyme inhibitor; HR, hazard ratio; CI, confidence interval; SLD, steatotic liver disease; MASLD, metabolic dysfunction-associated steatotic liver disease.

Supplementary Table S8. Comparison of overall survival between ARB and ACEI after IPTW: subgroup analyses by sex, age, body mass index, and type 2 diabetes.

Cohort	Overall survival			
	Female		Male	
	HR (CI 95%)	*P* value	HR (CI 95%)	*P* value
**Entire cohort**	0.93 (0.86, 1.01)	0.065	0.95 (0.90, 1.01)	0.133
**No SLD**	0.92 (0.78, 1.09)	0.290	1.02 (0.84, 1.23)	0.861
**MASLD**	0.94 (0.85, 1.03)	0.129	0.95 (0.89, 1.01)	0.090

Cohort	Overall survival			
	Age < 65		Age ≥ 65	
	HR (CI 95%)	*P* value	HR (CI 95%)	*P* value
**Entire cohort**	0.96 (0.90, 1.03)	0.302	0.93 (0.87, 1.00)	0.049
**No SLD**	1.05 (0.88, 1.26)	0.571	0.86 (0.72, 1.03)	0.103
**MASLD**	0.95 (0.88, 1.02)	0.162	0.94 (0.88, 1.02)	0.117

Cohort	Overall survival			
	BMI < 25		BMI ≥ 25	
	HR (CI 95%)	*P* value	HR (CI 95%)	*P* value
**Entire cohort**	0.96 (0.85, 1.09)	0.553	0.94 (0.89, 0.99)	0.021
**No SLD**	0.97 (0.84, 1.12)	0.700	0.89 (0.70, 1.13)	0.336
**MASLD**	0.94 (0.77, 1.16)	0.603	0.94 (0.90, 1.00)	0.038

Cohort	Overall survival			
	No diabetes		Diabetes	
	HR (CI 95%)	*P* value	HR (CI 95%)	*P* value
**Entire cohort**	0.93 (0.87, 0.99)	0.024	0.97 (0.90, 1.04)	0.363
**No SLD**	0.92 (0.80, 1.06)	0.223	0.97 (0.74, 1.28)	0.843
**MASLD**	0.93 (0.87, 1.00)	0.047	0.97 (0.90, 1.04)	0.377

ARB, angiotensin receptor blocker; ACEI, angiotensin-converting enzyme inhibitor; IPTW, inverse probability of treatment weighting; HR, hazard ratio; CI, confidence interval; SLD, steatotic liver disease; MASLD, metabolic dysfunction-associated steatotic liver disease; BMI, body mass index.

Supplementary Table S9. Comparison of cardiovascular events between ARB and ACEI after IPTW: subgroup analyses by sex, age, body mass index, and type 2 diabetes.

Cohort	Risk of cardiovascular event			
	Female		Male	
	HR (CI 95%)	*P* value	HR (CI 95%)	*P* value
**Entire cohort**	1.00 (0.94, 1.06)	0.927	0.88 (0.84, 0.92)	< 0.001
**No SLD**	1.01 (0.89, 1.14)	0.876	0.92 (0.80, 1.04)	0.216
**MASLD**	1.00 (0.94, 1.07)	0.986	0.87 (0.84, 0.91)	< 0.001

Cohort	Risk of cardiovascular event			
	Age < 65		Age ≥ 65	
	HR (CI 95%)	*P* value	HR (CI 95%)	*P* value
**Entire cohort**	0.90 (0.86, 0.94)	< 0.001	0.97 (0.92, 1.02)	0.221
**No SLD**	0.90 (0.80, 1.02)	0.108	1.00 (0.87, 1.15)	0.983
**MASLD**	0.90 (0.85, 0.94)	< 0.001	0.96 (0.90, 1.01)	0.123

Cohort	Risk of cardiovascular event			
	BMI < 25		BMI ≥ 25	
	HR (CI 95%)	*P* value	HR (CI 95%)	*P* value
**Entire cohort**	0.89 (0.81, 0.97)	0.013	0.93 (0.89, 0.96)	< 0.001
**No SLD**	0.92 (0.83, 1.03)	0.175	0.96 (0.82, 1.13)	0.630
**MASLD**	0.81 (0.69, 0.94)	0.011	0.92 (0.89, 0.96)	< 0.001

Cohort	Risk of cardiovascular event			
	No diabetes		Diabetes	
	HR (CI 95%)	*P* value	HR (CI 95%)	*P* value
**Entire cohort**	0.89 (0.85, 0.93)	< 0.001	0.98 (0.93, 1.04)	0.496
**No SLD**	0.92 (0.83, 1.01)	0.087	1.00 (0.81, 1.24)	0.994
**MASLD**	0.88 (0.84, 0.92)	< 0.001	0.98 (0.93, 1.04)	0.505

ARB, angiotensin receptor blocker; ACEI, angiotensin-converting enzyme inhibitor; IPTW, inverse probability of treatment weighting; HR, hazard ratio; CI, confidence interval; SLD, steatotic liver disease; MASLD, metabolic dysfunction-associated steatotic liver disease; BMI, body mass index.

Supplementary Table S10. IPTW-adjusted comparison of overall survival between ARB and ACEI across subgroups defined by hepatic fibrosis score.

Cohort	Overall survival			
	FIB-4 score < 1.45		FIB-4 score ≥ 1.45	
	HR (CI 95%)	*P* value	HR (CI 95%)	*P* value
**Entire cohort**	0.91 (0.85, 0.97)	0.006	0.98 (0.92, 1.05)	0.658
**No SLD**	0.98 (0.81, 1.19)	0.847	0.90 (0.76, 1.07)	0.239
**MASLD**	0.90 (0.83, 0.96)	0.003	1.00 (0.93, 1.08)	0.986
	**Overall survival**			
**Cohort**	**NFS < -1.455**		**NFS ≥ -1.455**	
	**HR (CI 95%)**	***P* value**	**HR (CI 95%)**	***P* value**
**Entire cohort**	0.89 (0.82, 0.97)	0.006	0.97 (0.92, 1.03)	0.379
**No SLD**	0.91 (0.77, 1.08)	0.274	0.96 (0.80, 1.16)	0.702
**MASLD**	0.88 (0.80, 0.97)	0.011	0.97 (0.92, 1.04)	0.417

ARB, angiotensin receptor blocker; ACEI, angiotensin-converting enzyme inhibitor; IPTW, inverse probability of treatment weighting; HR, hazard ratio; CI, confidence interval; SLD, steatotic liver disease; MASLD, metabolic dysfunction-associated steatotic liver disease; FIB-4, fibrosis-4; NFS, NAFLD fibrosis score.

Supplementary Table S11. IPTW-adjusted comparison of cardiovascular events between ARB and ACEI across subgroups defined by hepatic fibrosis score.

Cohort	Risk of cardiovascular event			
	FIB-4 score < 1.45		FIB-4 score ≥ 1.45	
	HR (CI 95%)	*P* value	HR (CI 95%)	*P* value
**Entire cohort**	0.94 (0.90, 0.99)	0.012	0.90 (0.86, 0.95)	< 0.001
**No SLD**	1.00 (0.87, 1.15)	0.964	0.89 (0.79, 1.00)	0.056
**MASLD**	0.93 (0.89, 0.98)	0.004	0.90 (0.85, 0.95)	< 0.001

Cohort	Risk of cardiovascular event			
	NFS < -1.455		NFS ≥ -1.455	
	HR (CI 95%)	*P* value	HR (CI 95%)	*P* value
**Entire cohort**	0.93 (0.88, 0.98)	0.009	0.92 (0.88, 0.96)	< 0.001
**No SLD**	0.97 (0.86, 1.09)	0.598	0.88 (0.77, 1.01)	0.071
**MASLD**	0.91 (0.86, 0.97)	0.004	0.92 (0.88, 0.97)	0.001

ARB, angiotensin receptor blocker; ACEI, angiotensin-converting enzyme inhibitor; IPTW, inverse probability of treatment weighting; HR, hazard ratio; CI, confidence interval; SLD, steatotic liver disease; MASLD, metabolic dysfunction-associated steatotic liver disease; FIB-4, fibrosis-4; NFS, NAFLD fibrosis score.
